# Adaptive restoration of T-cell motility by cold acclimation through metabolic and transcriptional remodeling

**DOI:** 10.3389/fimmu.2026.1789182

**Published:** 2026-04-10

**Authors:** Masahiro Hattori, Noriyoshi Onishi, Tatsuya Sato, Takashi Nakakura, Takeshi Suzuki

**Affiliations:** 1 Department of Biology, Center for Medical Education, Sapporo Medical University,Sapporo, Japan; 2 Division of Cellular Physiology and Signal Transduction, Department of Physiology, Sapporo Medical University School of Medicine, Sapporo, Japan; 3 Department of Anatomy, Teikyo University School of Medicine, Tokyo, Japan

**Keywords:** cell motility, cold acclimation, mitochondrial respiration, spare respiratory capacity, T cells, temperature adaptation, transcriptomic remodeling

## Abstract

**Objectives:**

Low temperatures reduce T-cell motility; however, the mechanisms by which T cells adapt to low-temperature environments remain poorly understood. Here, we investigated how T cells respond and adapt to subphysiological temperatures.

**Methods:**

Conalbumin-specific D10 T cells were analyzed by time-lapse live-cell imaging using an inverted microscope equipped with a temperature-controlled chamber. Cellular respiration was assessed using a Seahorse XFe96 analyzer, and transcriptomic changes were examined by RNA sequencing.

**Results:**

At 37°C, T cells exhibited active lamellipodia-driven migration. Reducing the temperature below 32°C progressively suppressed crawling motility, accompanied by increased cell rounding. At 26°C, motility was markedly reduced in non-acclimated T cells. Unexpectedly, T cells transiently exposed to low temperatures during routine passaging retained motility under cold conditions, suggesting the induction of cold acclimation. Consistent with this interpretation, T cells cultured at temperatures between 30°C and 26°C for 24 h exhibited enhanced motility when evaluated at the corresponding temperatures compared with non-acclimated cells, with the most pronounced functional difference observed at 26°C. Cold acclimation at 26°C was also associated with a marked increase in mitochondrial spare respiratory capacity. RNA sequencing revealed extensive transcriptional reprogramming following cold acclimation, characterized by upregulation of pathways related to ribosome biogenesis, RNA processing, transcriptional regulation, and protein quality control, and downregulation of gene sets involved in DNA replication, DNA repair, and cell cycle progression, consistent with adaptive cellular remodeling.

**Conclusion:**

These findings demonstrate that T-cell motility is highly temperature-sensitive, but prior exposure to low temperature enables adaptive restoration of migratory activity through coordinated metabolic and transcriptional remodeling. This adaptive response suggests that temperature actively regulates immune cell dynamics rather than serving solely as an environmental constraint and provides new insight into temperature-dependent immune regulation.

## Introduction

T cells are essential components of adaptive immunity, recognizing antigens presented by antigen-presenting cells and executing effector functions ranging from cytokine production to cytotoxic activity (1–3). These processes require dynamic cellular behavior, including continuous migration and cytoskeletal remodeling. A key aspect of T-cell activation is the formation of the immunological synapse, a specialized interface that coordinates T cell receptor (TCR) signaling and adhesion. To efficiently engage antigen-presenting cells, T cells must retain motility and dynamically extend lamellipodia and filopodia to survey their surroundings (4–6).

It is increasingly recognized that immune cell function is strongly shaped by the local microenvironment, including oxygen tension, nutrient availability, and metabolic constraints (7, 8). In contrast, temperature has received comparatively little attention. Although mammals tightly regulate core body temperature, immune cells are frequently exposed to lower temperatures. Fever transiently elevates tissue temperature (9, 10), whereas inflammation, ischemia, or tissue injury can locally reduce temperature. Clinically, systemic hypothermia is intentionally induced during cardiac surgery or organ transplantation (11, 12), and immune cells stored under hypothermic conditions may re-enter the circulation following transplantation (13). Incidental or therapeutic hypothermia in critical care settings likewise exposes circulating lymphocytes to cold environments.

Beyond these clinical contexts, immune cells in peripheral tissues are chronically subjected to subphysiological temperatures. For example, skin temperature at the fingertips is typically several degrees lower than core body temperature and decreases further during winter (14, 15). During outdoor cold exposure, fingertip temperatures can fall below 10°C within minutes, although this decline is partially counteracted by cold-induced vasodilation (16, 17). These observations underscore that lymphocytes operating in distal tissues must preserve at least basal functional activity in environments substantially colder than 37°C.

Early studies suggested that hypothermia broadly suppresses immune responses, consistent with the increased susceptibility to infection observed in hypothermic patients (18–21). However, the mechanisms underlying this suppression remain poorly defined. In particular, the effects of temperature on T-cell motility and cytoskeletal remodeling have not been systematically investigated. Whether T cells can adapt to sustained cold exposure—via a process referred to here as cold acclimation—remains largely unknown. In other cell types, such as neurons, cold exposure induces synaptic remodeling and promotes survival through RBM3-dependent mechanisms (22). More broadly, cold adaptation in mammalian cells is associated with alterations in membrane properties, cytoskeletal organization, gene expression, and metabolic remodeling (23, 24).

Cellular metabolism represents another critical regulator of T cell function. Upon activation, T cells undergo intrinsic metabolic reprogramming, engaging glycolysis and mitochondrial respiration to sustain effector responses (25, 26). However, the extent to which these metabolic pathways are sensitive to temperature changes remains unclear. Addressing this issue may provide fundamental insights into how immune cells respond to environmental stresses such as low temperature.

Despite the frequent exposure of immune cells to subphysiological temperatures in peripheral tissues, it remains largely unknown how such cold environments influence T cell behavior. Specifically, whether T cells merely experience passive functional suppression or actively adapt to maintain motility, cytoskeletal dynamics, and metabolic activity while traversing cold tissues has not been systematically examined. Addressing this gap is essential for understanding how immune surveillance is preserved in cold-exposed tissues and may uncover coordinated metabolic and transcriptional mechanisms underlying temperature-dependent adaptation in T cells.

## Materials and methods

### Cells and culture

The cloned helper T cell line D10.G4.1 (D10), which recognizes chicken conalbumin peptide presented by I-A^k^ (27), and the murine B lymphoma line CH12 (28) were kindly provided by Dr. A. Kupfer. Throughout this study, D10 cells are referred to as “T cells” unless otherwise specified. Cells were maintained at 37°C in a humidified incubator with 5% CO_2_. D10 cells were maintained in Dulbecco’s Modified Eagle Medium (DMEM; 08458-16, Nacalai Tesque Inc., Kyoto, Japan) supplemented with 10% fetal bovine serum, penicillin (100 U/ml), streptomycin (100 μg/ml), L-glutamine (2 mM), non-essential amino acids, and IL-2 (20 U/ml). CH12 cells were also maintained with the same medium except without IL-2. To minimize unintended cold exposure during routine handling, culture media used for passaging were prewarmed to 37°C prior to use.

### Cold acclimation and acute temperature shift protocols

For cold acclimation, T cells were incubated at 26°C for 24 h in a temperature-controlled incubator prior to functional analyses. For acute temperature shift experiments, cells maintained at 37°C were transferred to pre-equilibrated aluminum block incubators set to the designated imaging temperature (22–34°C or 26°C, as indicated). A volume of 100–200 µL of cell suspension was placed in 15 mL tubes and pre-equilibrated for approximately 5 min prior to transfer to glass-bottom dishes for imaging. Antigen-presenting cells were pre-equilibrated separately under identical conditions.

### Cell viability and proliferation analysis

Cell viability was assessed using trypan blue exclusion assay. Cells were collected at 0 h and after 24 h incubation at 37°C or 26°C, mixed with 0.4% trypan blue solution, and counted using a hemocytometer. Viable cells were defined as trypan blue–negative cells and expressed as a percentage of total cell number. Cell proliferation over 24 h was calculated as the fold change in total viable cell number relative to baseline (0 h). Experiments were performed using three independent biological replicates, and for each replicate, cell counts were conducted in quadruplicate prior to statistical analysis.

### Live-cell imaging

Live-cell imaging was performed using an Olympus IX81 inverted microscope (Olympus, Tokyo, Japan) equipped with a temperature-controlled incubation system. To prevent temperature hunting (oscillatory deviation from the set temperature) and to minimize external disturbances, the entire optical setup, including the microscope body, was enclosed within a custom-made acrylic housing. The temperature within the chamber was stabilized for at least 30 min prior to imaging, and fluctuations during acquisition were maintained within ±0.2°C of the set temperature. Humidified 5% CO_2_ was supplied by passing the gas through a water bottle placed inside the incubation chamber, and then directed to the culture dish via the dish lid to maintain appropriate CO_2_ concentration and humidity. Glass-bottom dishes (D11130H, Matsunami Glass Ind. Ltd., Osaka, Japan) were used for imaging. T cells (70–150 μL) were first placed onto the glass coverslip area and mounted on the microscope. After focus was adjusted, an equal volume of CH12 cells was added, after which time-lapse acquisition was initiated. For antigen-specific interaction assays, CH12 cells had been pretreated with 20 mg/mL conalbumin for 15–24 h prior to imaging. Immediately before imaging, both T cells and CH12 cells were concentrated 4–5-fold by centrifugation at 300 × g for 5 min, followed by removal of the supernatant. Concentrated cells were maintained at either the acclimation temperature or 37°C in a humidified 5% CO_2_ atmosphere until imaging. The final plating density was adjusted as needed based on imaging quality. Time-lapse images were acquired at 20-s intervals for 60 consecutive time points (total imaging time, 20 min) using a 40× Plan Achromat objective lens (numerical aperture, 0.85). At each time point, images were acquired at multiple focal planes spanning a 2-μm range in the z-axis to compensate for vertical displacement of floating cells and to ensure continuous acquisition of in-focus images. Images were captured with a CoolSNAP MYO camera (Photometrics, Tucson, AZ, USA) mounted on the microscope, and acquisition was controlled using SlideBook 5.0 software (3i Intelligent Imaging Innovations, Denver, CO, USA), which managed time-lapse acquisition, z-stack scanning, and camera triggering.

### Analysis of T-cell motility and morphology

Time-lapse images of T cells were acquired at 20-s intervals for 60 time points (total duration, 20 min). For quantitative analysis, cells were selected based on predefined objective criteria. Only cells that remained within the field of view throughout the entire recording period, were sufficiently separated to allow accurate segmentation, and did not overlap with neighboring cells were included. TIFF image series were generated, and cells meeting these criteria were selected for analysis. Cell contours were segmented using the Object Selection Tool in Adobe Photoshop (Adobe Systems, San Jose, CA, USA), and binary mask images were generated. The cell area, perimeter, centroid coordinates, and circularity index were measured using ImageJ software (National Institutes of Health, Bethesda, MD, USA). Centroid displacement and cell deformation area were calculated at 3-min intervals. Let $C_{t}$ and $C_{t+1}$ denote the binarized cell regions at two time points separated by 3 min. The cell deformation area ( $A_{CD}$) was defined as:


$$A_{CD}=\text{Area}\left(C_{t+1}\setminus C_{t}\right)$$

which corresponds to the cellular area newly occupied at time t+1 relative to time t. This value was calculated after superimposing the segmented binary masks. In purely translational movement, the newly gained area is expected to be comparable to the area lost between frames, whereas asymmetric changes reflect spreading or contraction. Thus, this parameter quantifies geometric remodeling of the cell contour between consecutive time points. A schematic illustration of this geometrical definition is provided in **Supplementary Figure S1**. The circularity index was calculated using the following formula:


$$\text{Circularity index }=\;(\text{perimete}{\text{r}}^{2})\;/\;(4\text{π }\times \text{ cell area)}$$

Each temperature condition was evaluated in a separate experimental session conducted on different days using independently prepared cell batches. Quantitative datasets shown represent one representative experimental session per condition, although similar temperature-dependent trends were reproducibly observed across independent preparations. Similar temperature-dependent trends were reproducibly observed across independent preparations. Ten non-overlapping cells per condition were analyzed for centroid displacement and cell deformation area (n = 10), and fifteen cells per condition were analyzed for circularity index (n = 15). Each data point shown in the figures represents a single-cell measurement. Cell selection was performed prior to analysis based solely on segmentation feasibility and tracking continuity, without reference to motility outcomes. Quantitative data are presented as mean ± SD. Statistical comparisons between two groups were performed using Welch’s t-test. Statistical significance was defined as follows: *p< 0.05, **p< 0.01, ***p< 0.001, and ****p< 0.0001.

### Extracellular flux assay

T cells were maintained at 37°C under physiological culture conditions and cold-acclimated at 26°C for 24 h. Prior to the assay, the culture medium was replaced with Seahorse XF DMEM assay medium (#103575-100, Agilent Technologies, Santa Clara, CA, USA) supplemented with 5.5 mM glucose, 2.0 mM glutamine, 1.0 mM sodium pyruvate, and 5.0 mM HEPES (pH 7.40). Non–cold-acclimated and cold-acclimated cells were seeded into poly-D-lysine–coated Seahorse XF96 cell culture microplates (#103794-100, Agilent Technologies) at 20,000 cells/well and incubated in a CO_2_-free incubator at 26°C for 45 min to allow temperature equilibration. Measurements were performed at 26°C to match the acclimation temperature. The oxygen consumption rate (OCR) and extracellular acidification rate (ECAR) were measured using a Seahorse XFe96 Analyzer (Agilent Technologies) with cycles of 3 min mixing followed by 3 min measurement. After basal measurements, cells were sequentially injected with oligomycin (2.0 µM; #75351, Sigma-Aldrich, St. Louis, MO, USA), carbonyl cyanide p-trifluoromethoxyphenylhydrazone (FCCP; 5.0 µM; #C2920, Sigma-Aldrich), and a rotenone/antimycin A mixture (1.0 µM; #R8875/#A8674, Sigma-Aldrich). Each condition was analyzed in five technical replicate wells within a single experimental set. Following the assay, cells in each well were lysed and total protein content was quantified using a BCA protein assay (Thermo Fisher Scientific, Waltham, MA, USA). OCR and ECAR values were normalized to total protein content per well. Basal respiration, maximal respiration, and spare respiratory capacity were calculated according to standard Seahorse XF analysis protocols. Time-course data are presented as mean ± SEM to improve visualization, whereas summary parameters are presented as mean ± SD. Statistical comparisons between non–cold-acclimated and cold-acclimated cells were performed on summary parameters using Welch’s t-test.

### RNA isolation and RNA sequencing

Total RNA was isolated from cold-acclimated T cells and non-acclimated control T cells using the RNeasy Mini Kit (74104; QIAGEN, Hilden, Germany). For each condition, three independently prepared biological replicates were used (n = 3). RNA sequencing was performed by Rhelixa, Inc. (Tokyo, Japan). RNA integrity was assessed by the provider, and samples that passed quality control criteria were used for library preparation. Poly(A)+ mRNA was isolated using the NEBNext Poly(A) mRNA Magnetic Isolation Module, and strand-specific sequencing libraries were constructed using the NEBNext Ultra II Directional RNA Library Prep Kit (New England Biolabs). Libraries were sequenced on a NovaSeq X Plus platform (Illumina, Inc., CA, USA) to generate paired-end reads of 150 bp (PE150), with a sequencing depth of approximately 20 million read pairs (40 million reads) per sample. Raw sequencing reads were subjected to quality control and aligned to the mouse reference genome GRCm39 (mm39) using HISAT2. Gene-level read counts were obtained using featureCounts. Differential expression analysis between cold-acclimated and control T cells was performed using DESeq2, with normalization based on the median-of-ratios method implemented in the package. Differentially expressed genes were defined as those with an absolute log2 fold change ≥ 1 and an adjusted p-value (false discovery rate, FDR)< 0.05, with multiple testing correction performed using the Benjamini–Hochberg method. Principal component analysis (PCA) was conducted using variance-stabilized transformed count data generated by DESeq2. Volcano plots were generated using log2 fold change and adjusted p-values from DESeq2 results. Gene Ontology (GO) and KEGG pathway enrichment analyses were performed using the clusterProfiler R package with FDR-adjusted p-values< 0.05 considered statistically significant.

## Results

### T-cell motility is significantly reduced in cold environments

**Figure 1:**
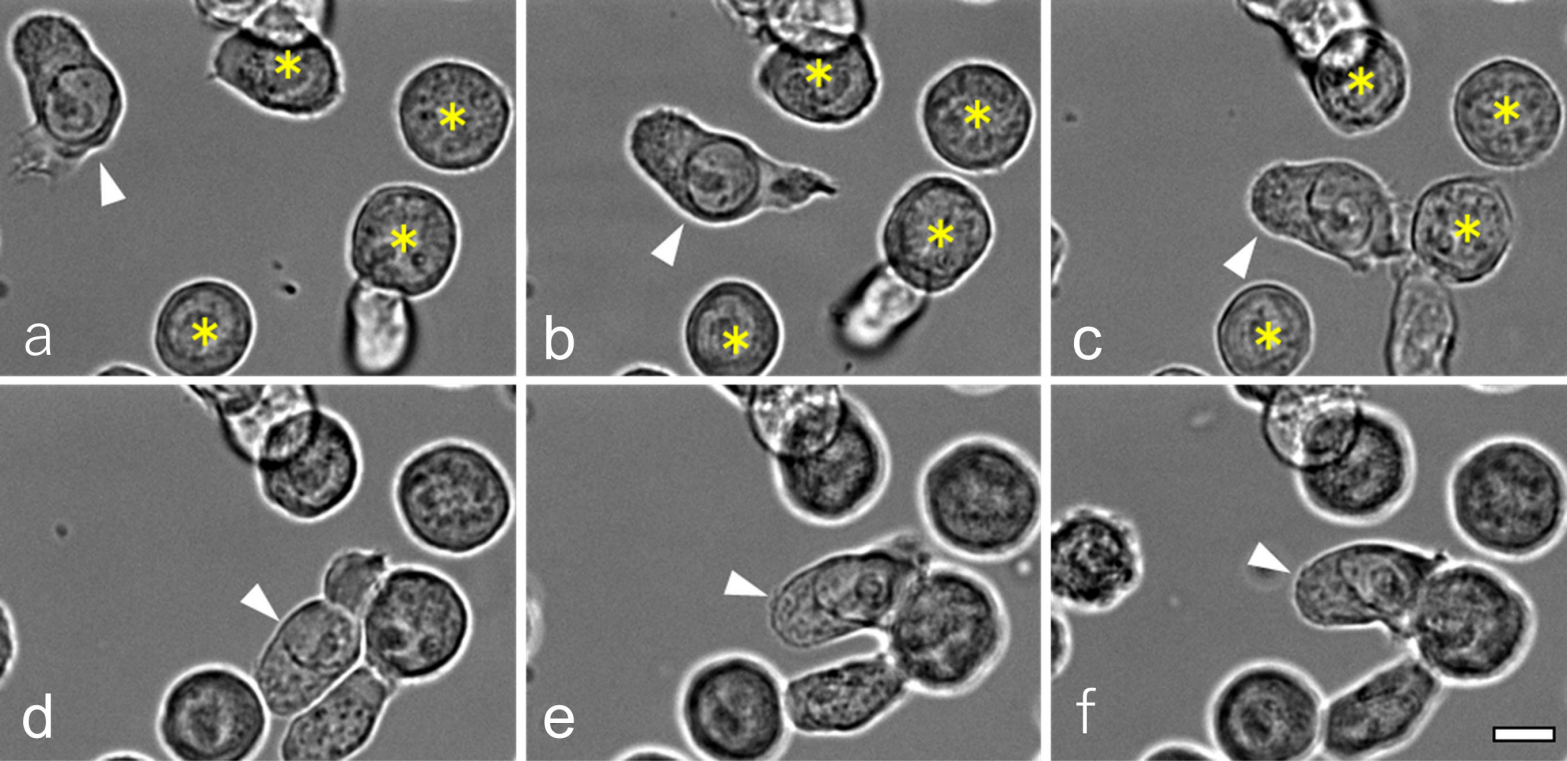
Dynamics of D10 T cells under physiological conditions (see also **Supplementary Video S2**). At 37°C, D10 T cells actively migrated across the glass-bottom dish using lamellipodia. Upon addition of antigen-presenting cells (APCs) bearing the cognate antigen, T cells searched for and contacted APCs, initiating stable cell–cell contact within a few minutes. Panels a–f are snapshots extracted at 2-min intervals from time-lapse images acquired every 5 s (shown in **Supplementary Video S2**), illustrating a representative sequence in which a migrating T cell encounters an APC and initiates stable cell–cell interaction. Arrowheads and asterisks (*) indicate a crawling T cell and APCs, respectively. Scale bar: 10 μm.

We analyzed T-cell motility at different temperatures using live-cell imaging. Under standard culture conditions at 37°C, T cells actively migrated across the glass-bottom dish by repeatedly extending and retracting lamellipodia. Representative full-field time-lapse recordings acquired at 20-s intervals for 20 min are shown in **Supplementary Video S1a**. Upon the addition of antigen-presenting cells (APCs) bearing cognate antigens, T cells efficiently searched for APCs, established stable T-cell–APC contact within a few minutes (**Figure 1a–f**; **Supplementary Video S2**).

**Figure 2:**
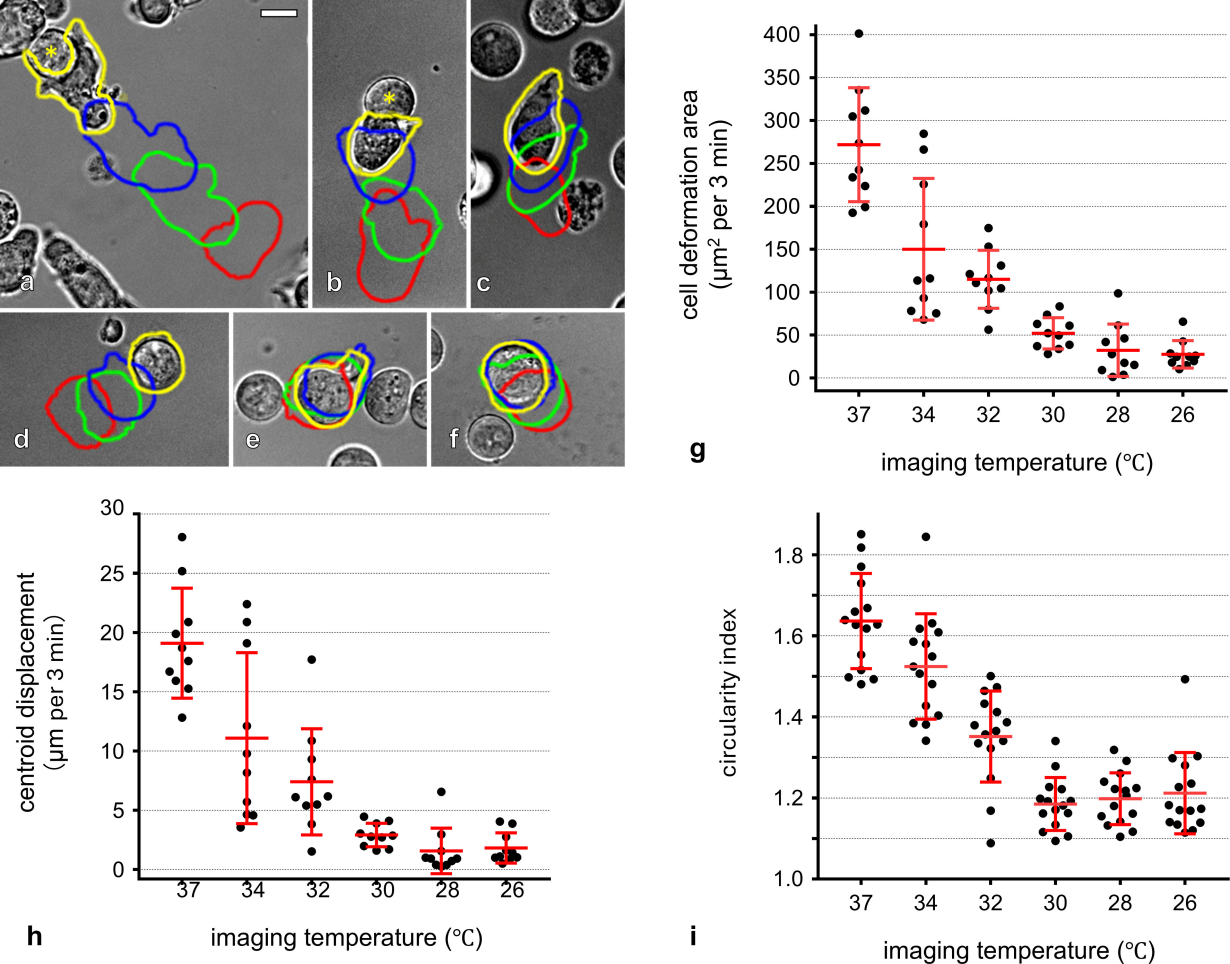
Effect of low temperature on T-cell motility and cell morphology (see also **Supplementary Video S3**). T cells maintained at 37°C were subjected to time-lapse imaging at 37°C **(a)**, 34°C **(b)**, 32°C **(c)**, 30°C **(d)**, 28°C **(e)**, and 26°C **(f)**. For each panel, cell outlines at 9, 6, and 3 min before the displayed frame and at the current time point are shown in red, green, blue, and yellow, respectively. Cell contours were automatically extracted using the object selection tool in Adobe Photoshop, and three parameters were quantified: cell deformation area **(g)**, defined as the area enclosed by the contour at time t+3 min that lies outside the contour at time t; and centroid displacement **(h)**, calculated as the migration length of the cell centroid over 3 min; and the circularity index **(i)**, calculated from the perimeter and area of each cell. Data in panels **(g–i)** are shown as mean ± SD. Each dot represents a single-cell measurement [n = 10 cells per condition for **(g, h)**; n = 15 cells per condition for **(i)**] obtained from a single independent experiment. Scale bar: 10 μm **(a–f)**. Asterisks (*) in **(a, b)** indicate antigen-presenting cells.

When the imaging temperature was lowered below 32°C, both crawling motility and membrane ruffling at the T-cell–APC interface progressively declined with decreasing temperature. At 26°C, T-cell movement was almost completely abolished, and stable T-cell–APC contact was rarely observed (**Figure 2a–f**; **Supplementary Video S1b**, **S3**). Quantitative analysis of time-lapse images revealed that both the cell deformation area and centroid displacement per 3 min—indices of morphological dynamics and cell locomotion—were significantly reduced at 26°C compared with those at 37°C (**Figure 2g, h**). Temperature reduction also markedly affected T-cell morphology. At physiological temperature, T cells exhibited a typical amoeboid shape characterized by dynamic lamellipodial protrusion and retraction. As the temperature decreased, these behaviors were progressively suppressed, and cells adopted a rounded and shrunken morphology (**Supplementary Video S3**). The proportion of rounded cells increased sharply below 32°C, and at 26°C, nearly all cells displayed this morphology. Consistently, quantification using the circularity index demonstrated a progressive increase in cell circularity with decreasing temperature (**Figure 2i**).

### Cold-acclimated T cells exhibit enhanced activity at low temperature

During routine subculture, we occasionally observed that when fresh medium was applied without prewarming, a subset of T cells retained detectable motility under transient cooling, although overall activity was reduced. This observation led us to hypothesize that prior exposure to low temperature attenuates the cold-induced suppression of T-cell motility. To test this hypothesis, T cells were precultured at 26°C for 24 h to induce cold acclimation, and their behavior was subsequently analyzed under reduced-temperature conditions.

**Figure 3:**
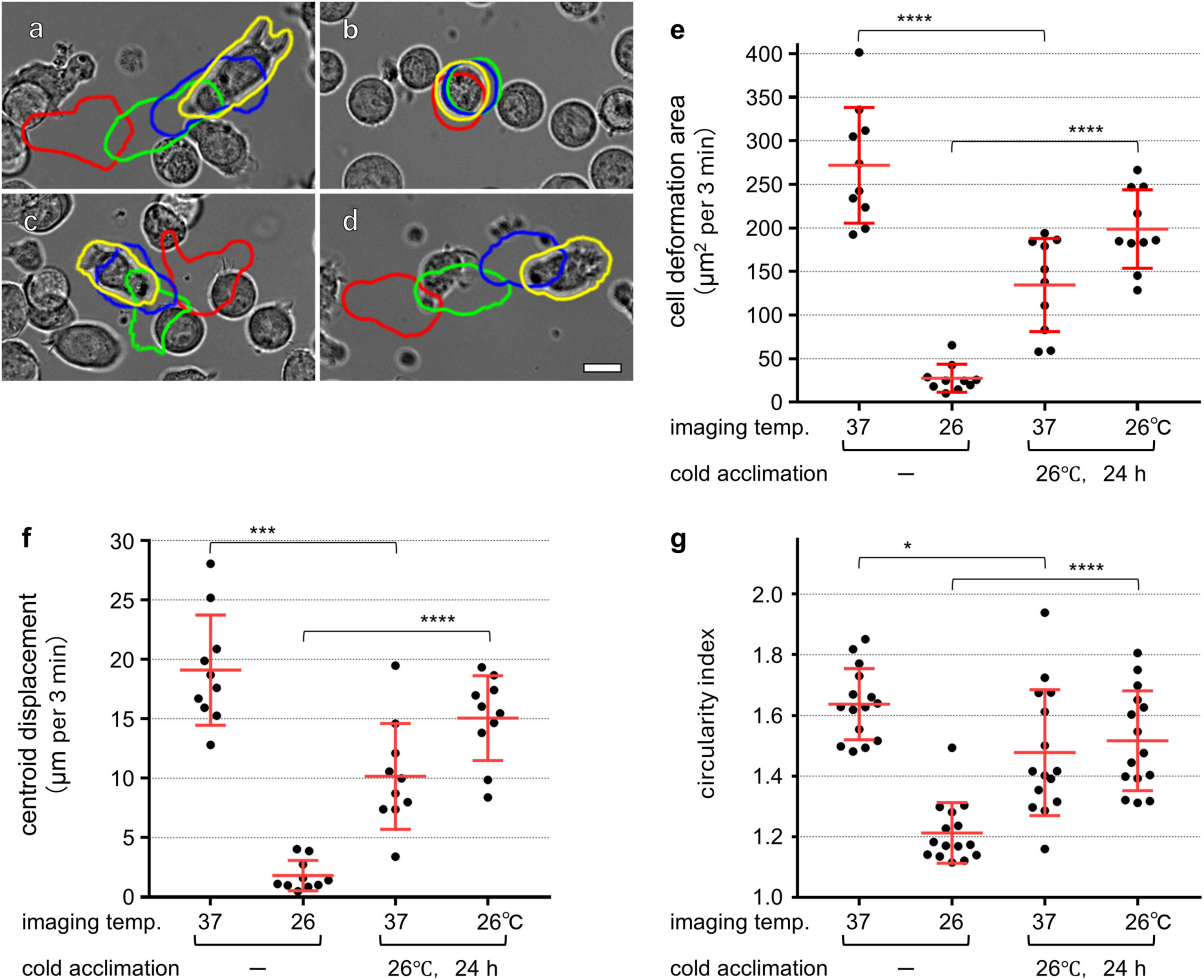
Activation of T-cell motility by cold acclimation (see also **Supplementary Video S4**). T cells maintained at 37°C were imaged either at 37°C **(a)** or acutely cooled to 26°C **(b)**, showing a marked reduction in motility at low temperature. In contrast, T cells acclimated to 26°C for 24 h displayed restored motility when imaged at 37°C **(c)** and maintained motility even when imaged at 26°C **(d)**. For each panel, cell outlines at 9, 6, and 3 min before the displayed frame and at the current time point are shown in red, green, blue, and yellow, respectively. Cell contours were extracted using the object selection tool in Adobe Photoshop. Three parameters were quantified: cell deformation area **(e)**, defined as the area extending beyond the contour obtained 3 min earlier; centroid displacement **(f)**, calculated as the migration length of the cell centroid over 3 min; and the circularity index **(g)**, calculated from the perimeter and area of each cell. Data in panels e–g are shown as mean ± SD, and statistical significance was assessed using Welch’s t-test. Each dot represents a single-cell measurement [n = 10 cells per condition for **(e, f)**; n = 15 cells per condition for **(g)**] obtained from a single independent experiment. Scale bar: 10 μm **(a–d)**. The difference between cold-acclimated cells imaged at 26°C vs 37°C was statistically significant for cell deformation area (**p< 0.01) and centroid displacement (*p< 0.05), although statistical markers are not shown to maintain visual clarity.

When non-acclimated T cells continuously maintained at 37°C were acutely cooled and imaged at 26°C, their motility was markedly suppressed, as indicated by diminished membrane ruffling and minimal centroid displacement (**Figures 3a, b**; **Supplementary Video S4**). In contrast, cold-acclimated T cells displayed robust crawling motility and active membrane ruffling at the cell–cell contact interface under the same low-temperature conditions (**Figures 3c, d**; **Supplementary Video S4**). Quantitative analysis revealed that the cell deformation area and centroid displacement per 3 min interval were increased by 7.21-fold and 8.28-fold, respectively, compared with non-acclimated cells imaged at 26°C (**Figures 3e, f**). Morphologically, cold-acclimated T cells retained an amoeboid shape with repeated lamellipodial extension and retraction at 26°C, whereas non-acclimated cells adopted a rounded morphology under the same conditions (**Figure 3g**). Notably, when cold-acclimated T cells were returned to 37°C, both motility parameters and the circularity index were significantly lower than those of non-acclimated T cells cultured at 37°C (**Figures 3e–g**). Furthermore, within the cold-acclimated group, cell deformation area and centroid displacement were significantly greater at 26°C than at 37°C, indicating that acclimation-induced enhancement of T-cell activity is most prominent under cold conditions.

To determine whether reduced temperature affects cell survival, we assessed cell viability using trypan blue exclusion assay before and after 24 h incubation at 37°C or 26°C (**Supplementary Figure S2**). Although viability showed a modest decrease after 24 h at 26°C, this change did not reach statistical significance (Welch’s t-test, p = 0.0808). In contrast, cell proliferation over 24 h was markedly suppressed at 26°C (1.066-fold increase) compared with 37°C (2.45-fold increase). These findings indicate that the functional suppression observed under acute cooling is not attributable to substantial loss of cell viability but is associated with reduced short-term proliferative expansion. In parallel with preserved viability, cell proliferation over 24 h was markedly reduced at 26°C compared with 37°C, indicating that subphysiological temperature strongly suppresses cell-cycle progression under our culture conditions.

### Enhanced mitochondrial respiratory activity in cold-acclimated T cells

**Figure 4:**
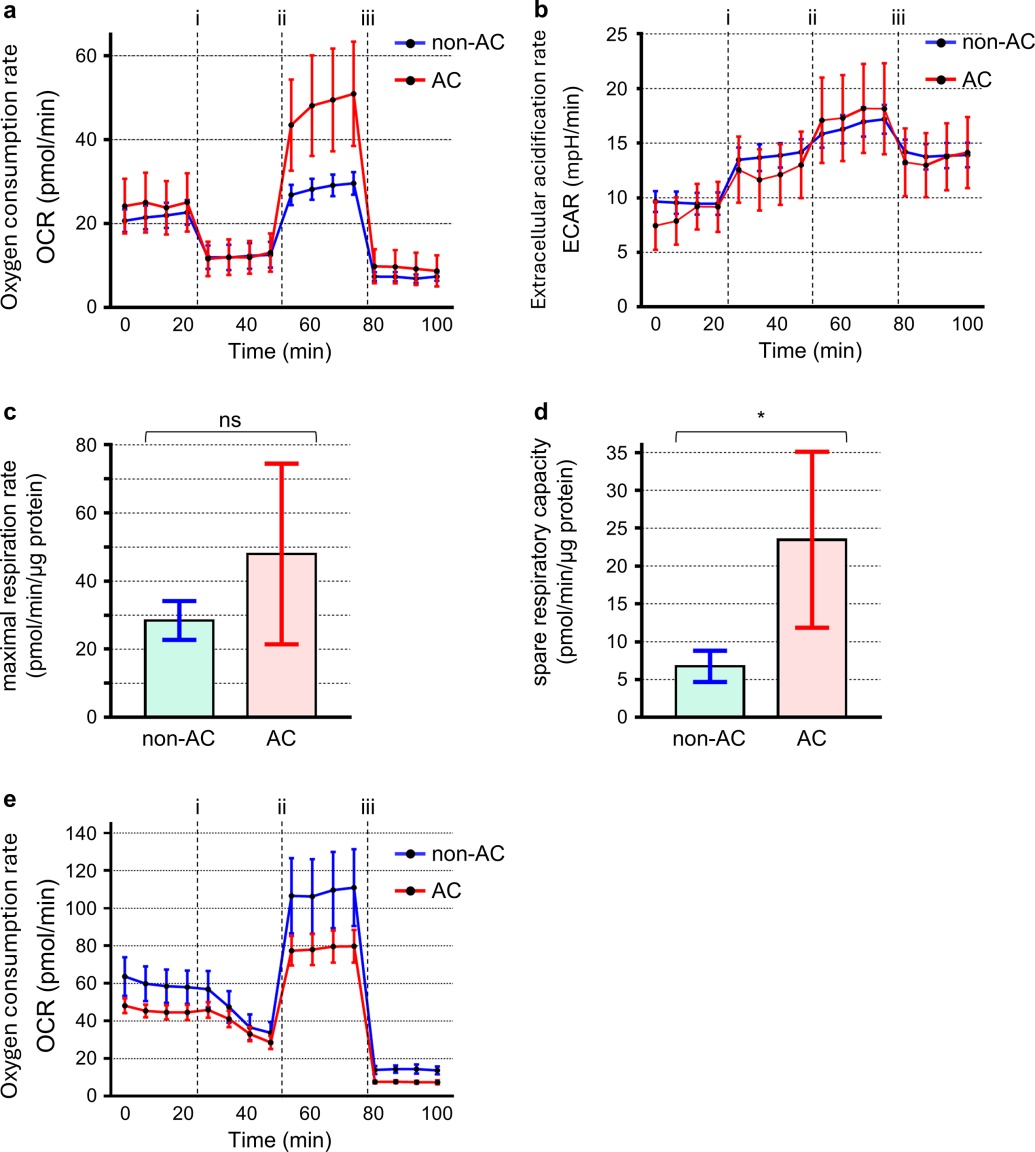
Effects of cold acclimation on mitochondrial and glycolytic function in T cells. T cells maintained at 37°C (non-AC) or acclimated to 26°C for 24 h (AC) were analyzed using a Seahorse XFe96 analyzer. Oxygen consumption rate (OCR, **(a)** and extracellular acidification rate (ECAR, **(b)** were monitored at baseline and after sequential injections of oligomycin (i), FCCP (ii), and rotenone/antimycin A (iii). Maximal respiration and spare respiratory capacity are shown in **(c, d)**, respectively. To determine whether respiratory differences persist under physiological temperature, OCR was also measured at 37°C **(e)**. Data in **(a, b, e)** represent mean ± SEM, whereas data in **(c, d)** represent mean ± SD. Statistical significance was assessed on summary parameters using Welch’s t-test. SEM is shown for time-course data **(a, b, e)** to indicate the precision of the mean across measurement cycles, whereas SD is shown for summary parameters **(c, d)** to reflect variability among experimental replicates. * p < 0.05; ns, not significant.

To determine whether cold acclimation enhances T-cell energy metabolism under low-temperature conditions, metabolic activity was assessed using a flux analyzer (**Figures 4a, b**). Under these conditions, cold-acclimated T cells exhibited a 1.69-fold increase in maximal respiration and a 3.49-fold increase in spare respiratory capacity compared with non-acclimated cells (**Figures 4c, d**). Although the increase in maximal respiration did not reach statistical significance, spare respiratory capacity was significantly elevated in cold-acclimated cells. In contrast, proton leak was comparable between the two groups, and glycolytic activity remained unchanged (**Figure 4b**), indicating that the enhanced respiratory phenotype was not accompanied by increased glycolysis. These results suggest that cold acclimation enhances mitochondrial oxidative capacity under low-temperature conditions. To examine whether this metabolic enhancement persists at physiological temperature, respiratory activity was also evaluated at 37°C (**Figure 4e**). Upon rewarming, non-acclimated cells displayed a pronounced increase in maximal respiration, whereas cold-acclimated cells showed little or no additional elevation relative to their low-temperature values. Consequently, the magnitude of temperature-dependent metabolic upregulation was reduced in cold-acclimated cells, indicating that these cells already possess elevated respiratory capacity prior to rewarming. As shown in (**Supplementary Figure S3**), extracellular acidification rate (ECAR) increased at 37°C in both groups, with a tendency toward higher values in cold-acclimated cells, indicating that glycolytic capacity is not impaired by prior cold acclimation.

### Temperature conditions required for cold acclimation of T cells

**Figure 5:**
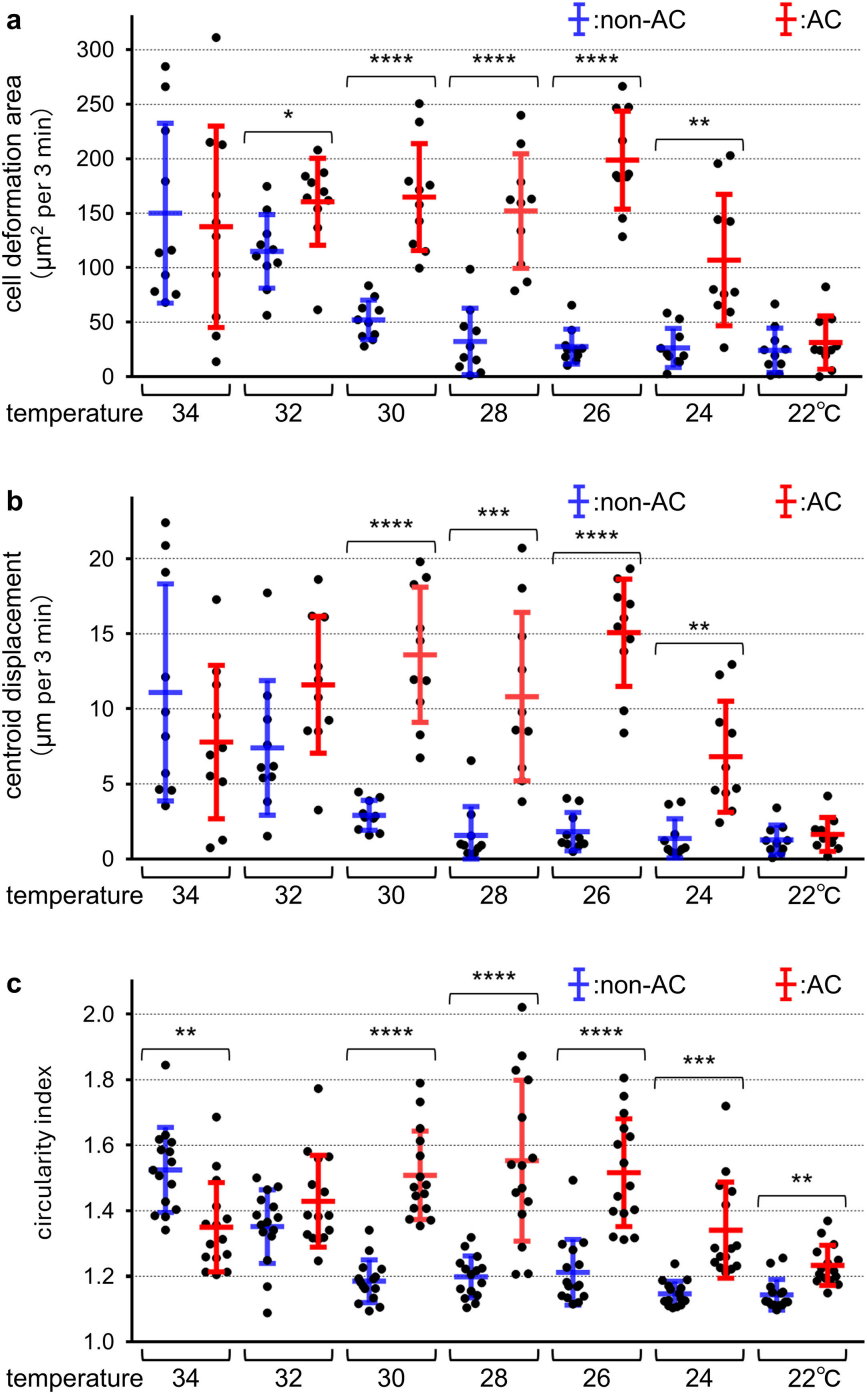
Temperature dependence of cold acclimation in T cells. T cells were incubated for 24 h at each indicated temperature (22–34°C) to induce cold acclimation and were subsequently imaged at the same temperature used during the incubation. Three motility-related parameters were quantified: cell deformation area **(a)**, defined as the area extending beyond the cell contour obtained 3 min earlier; centroid displacement **(b)**, defined as the migration length of the centroid over 3 min; and the circularity index **(c)**, calculated from cell perimeter and area. Blue bars represent non-acclimated cells maintained at 37°C before imaging (non-AC), and red bars represent cold-acclimated cells (AC). Data represent mean ± SD. Each dot represents a single-cell measurement [n = 10 cells per condition for **(a, b)**; n = 15 cells per condition for **(c)**] obtained from a single independent experiment. Asterisks indicate significant differences between non-AC and AC cells at each temperature (Welch’s t-test).

To define the temperature range required for cold acclimation, T cells maintained at 37°C were transferred to 34°C, 32°C, 30°C, 28°C, 26°C, or 22°C for 24 h, and their motility was assessed by live-cell imaging at the corresponding acclimation temperature. T cells precultured at 34°C displayed detectable crawling activity when imaged at 34°C; however, their motility was slightly reduced compared with that of non-acclimated cells examined at the same temperature (**Figures 5a–c**). Although changes in cell deformation area and centroid displacement did not reach statistical significance, the circularity index was significantly decreased at 34°C, indicating partial suppression of amoeboid morphology. At intermediate temperatures (32°C, 30°C, and 28°C), cold-acclimated cells consistently showed higher motility than non-acclimated cells when evaluated at the same temperature (**Figures 5a–c**), indicating that cold acclimation was induced across this temperature range of intermediate temperatures. Among the temperatures tested, acclimation at 26°C produced the largest difference between non-acclimated and acclimated cells, reflecting both a marked reduction in motility in non-acclimated cells and a robust enhancement in acclimated cells. This temperature therefore represents the condition under which the functional impact of cold acclimation is most clearly manifested. Cells acclimated at 24°C also showed significant improvements in motility-related parameters, although the magnitude of enhancement was lower than that observed at 26°C. Notably, acclimation at 22°C failed to improve either cell motility or morphology, suggesting that excessively low temperatures impair the cellular processes required for effective acclimation. Together, these results define a narrow temperature window for effective cold acclimation of T cells, with the maximal functional impact observed at approximately 26°C.

### Relationship between duration of cold exposure and cold acclimation in T cells

**Figure 6:**
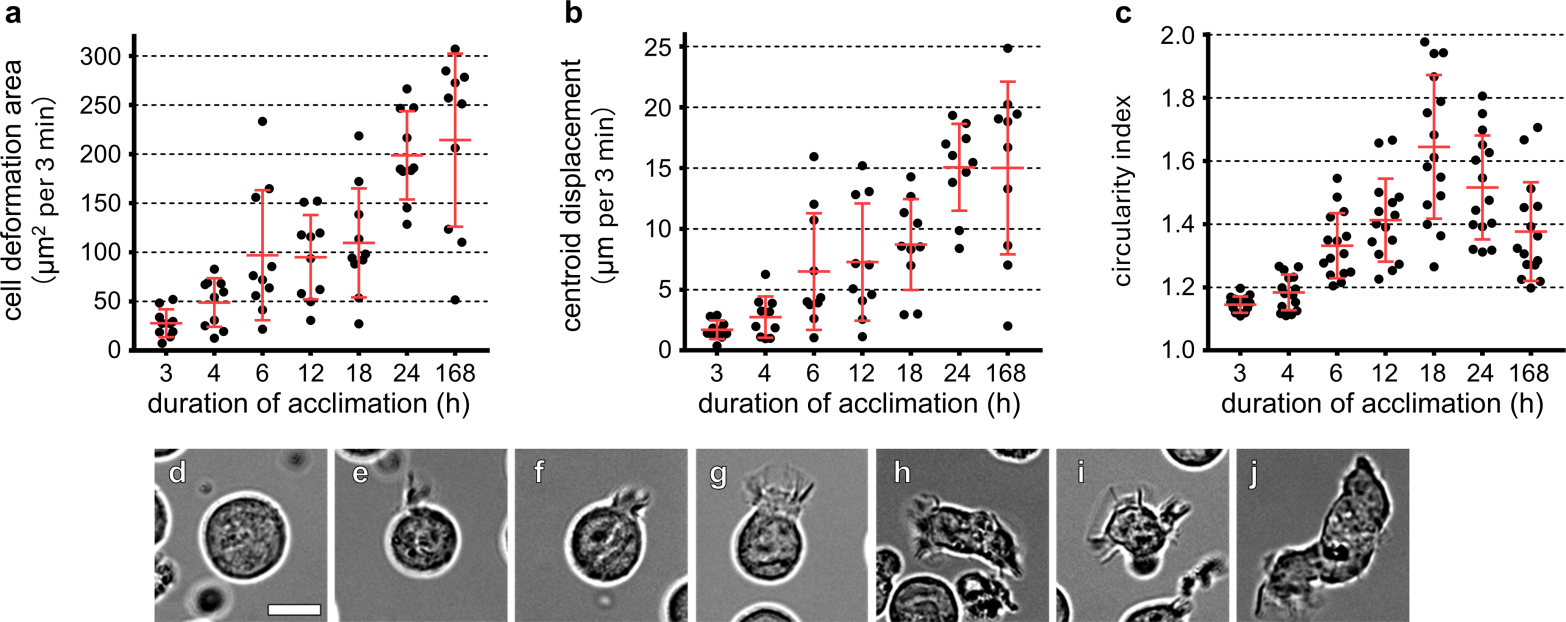
Relationship between the duration of cold exposure and cold acclimation in T cells (see also **Supplementary Video S5**). T cells maintained at 37°C were exposed to 26°C for 3, 4, 6, 12, 18, 24, or 168 h, and their motility and morphology were analyzed at 26°C. Cell deformation area **(a)**, centroid migration length **(b)**, and the circularity index **(c)** were quantified. Longer exposure to the cold environment progressively increased these parameters, indicating gradual acquisition of cold acclimation. **(d–j)** Show representative cell outlines, illustrating the progressive changes in cell morphology associated with cold acclimation. Data in **(a–c)** are presented as mean ± SD. Each dot represents a single-cell measurement [n = 10 cells per condition for **(a, b)**; n = 15 cells per condition for **(c)**] obtained from a single independent experiment. Scale bar: 10 μm **(d–j)**.

To determine the duration of cold exposure required for cold acclimation, T cells maintained at 37°C were transferred to 26°C and analyzed at multiple time points by live-cell imaging at 26°C. When cells were exposed to 26°C for ≤3 h, they remained rounded and contracted and exhibited virtually no motility, resembling non-acclimated control cells (**Figures 6a–d**; **Supplementary Video S5**). After 4 h of exposure, most cells still displayed an inactive morphology; however, a small subset began to extend and retract fine filopodia from restricted regions of the plasma membrane (**Figures 6a–c, e**; **Supplementary Video S5**). With increasing duration of cold exposure, both the proportion of cells exhibiting protrusive activity and the spatial extent of membrane remodeling progressively increased (**Figures 6d–j**; **Supplementary Video S5**). By 18 h, many cells showed dynamic extension and retraction of both filopodia and lamellipodia across the entire cell perimeter and resumed active crawling behavior. Cell motility reached near-maximal levels after 24 h of cold exposure, and further incubation at 26°C did not produce additional enhancement. Together, these results indicate that cold acclimation develops gradually and requires approximately 18–24 h of sustained cold exposure for full manifestation.

### Changes in gene expression of T cells induced by cold acclimation

**Figure 7:**
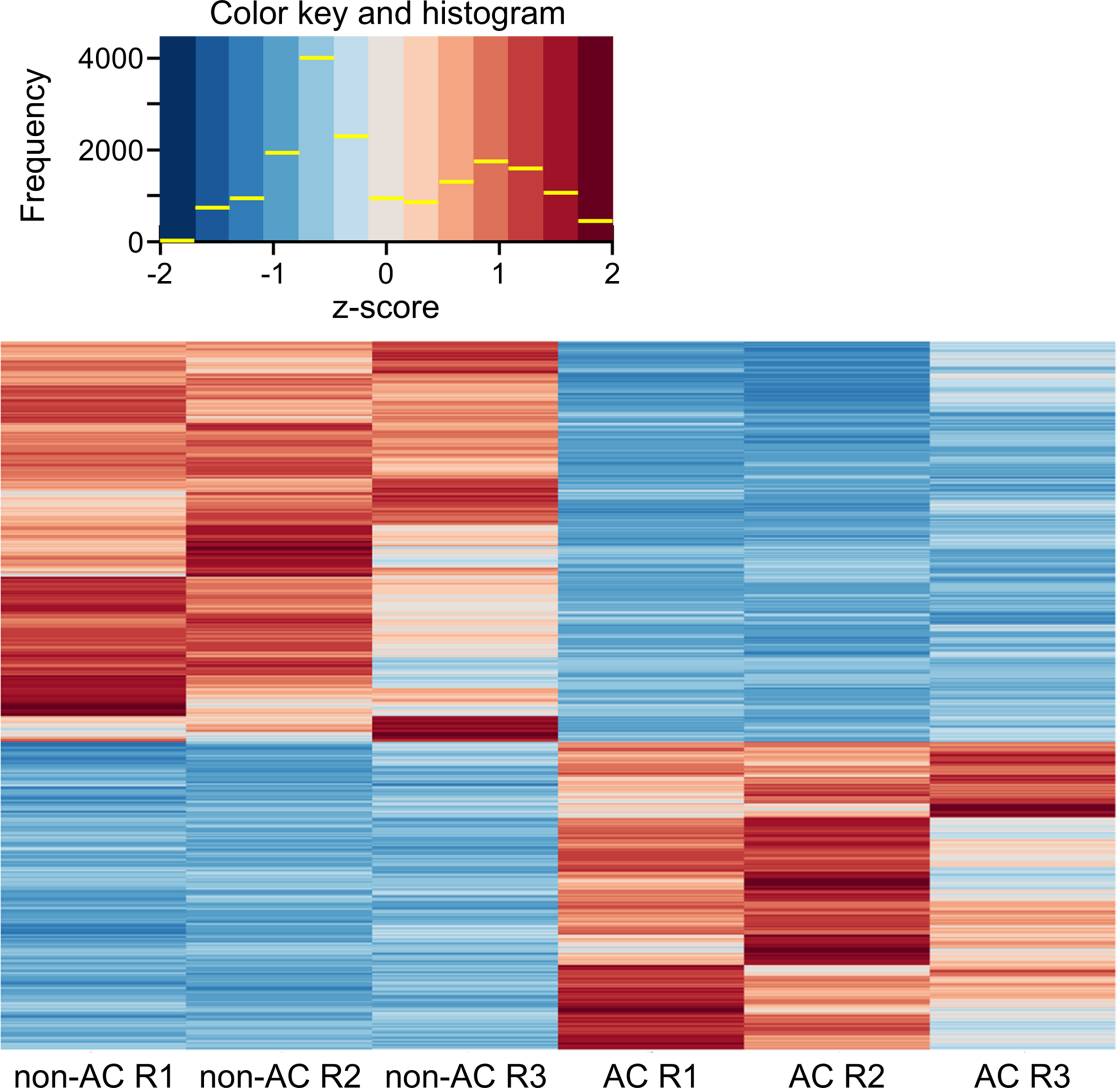
Heatmap showing relative expression levels of differentially expressed genes between non-acclimated (non-AC) and cold-acclimated (AC; 26°C for 24 h) T cells. For each condition, three independently prepared biological replicates were analyzed (R1–R3). Expression values were row-scaled and are shown as Z-scored values. The color key and histogram (top) indicate the distribution of Z-scored expression values used for heatmap visualization, ranging from −2 to +2. See also **Supplementary Figures S4**, **S5** for PCA and volcano plot analyses supporting the RNA-seq analysis, respectively.

To examine whether cold acclimation alters gene expression patterns in T cells, RNA sequencing analysis was performed using three independently prepared biological replicates (R1–R3). Principal component analysis (PCA) demonstrated tight clustering of replicates within each condition and clear separation between non-acclimated and cold-acclimated samples, indicating high reproducibility and a robust global transcriptional shift induced by cold acclimation (**Supplementary Figure S4**). Across all replicates, global gene expression profiles were markedly altered following cold acclimation at 26°C for 24 h (**Figure 7**). Of 15,234 detected genes, 1,243 (8.2%) were significantly upregulated and 1,008 (6.6%) were downregulated (FDR< 0.05, |log2FC| > 1). A volcano plot illustrating the distribution of differentially expressed genes further confirmed widespread transcriptional reprogramming (**Supplementary Figure S5**). Notably, genes that were highly expressed at 37°C were predominantly downregulated after cold acclimation (upper cluster), whereas genes with low basal expression at 37°C were upregulated (lower cluster). These reciprocal changes indicate a large-scale reorganization of gene expression programs in T cells in response to cold acclimation.

**Figure 8:**
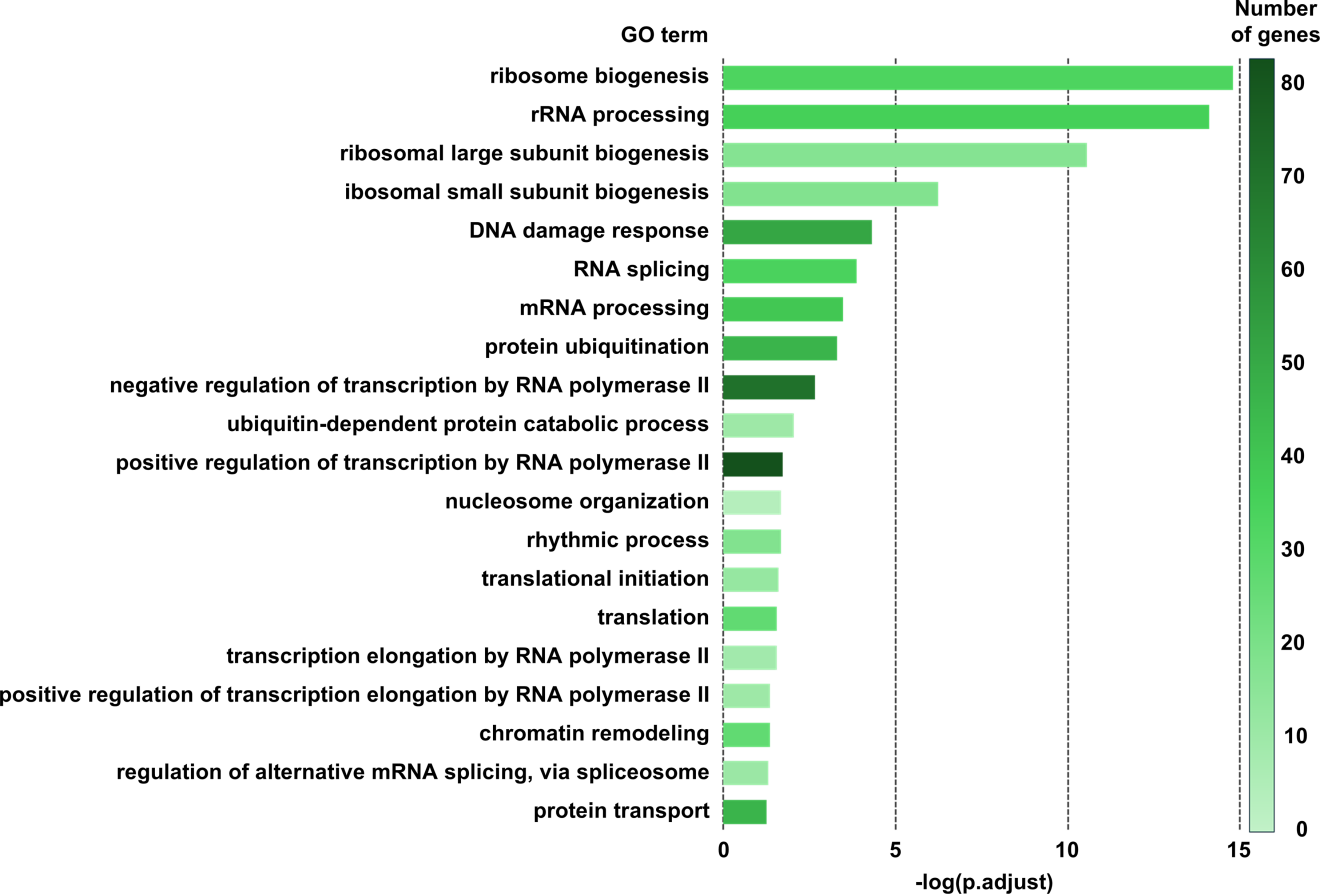
GO enrichment analysis (Biological Process) of genes upregulated in cold-acclimated T cells. The horizontal axis represents the −log10 of the adjusted p-values (FDR-corrected). Bar colors indicate the number of genes associated with each biological process. See also **Supplementary Figure S6** for KEGG pathway enrichment analysis.

Gene Ontology (GO) enrichment analysis further revealed that cold acclimation induces coordinated remodeling of cellular pathways associated with adaptive responses. Genes related to nuclear RNA regulation—including ribosome biogenesis, translational initiation, RNA splicing, and RNA processing—as well as chromatin organization, transcriptional regulation, and protein quality control processes such as ubiquitination were significantly upregulated in cold-acclimated T cells (**Figure 8**). KEGG pathway enrichment analysis yielded results consistent with the GO analysis, further supporting coordinated regulation of ribosome biogenesis, protein quality control, and metabolic pathways (**Supplementary Figure S6**). In contrast, gene clusters associated with DNA replication and repair, cell cycle progression, immune and stress responses, and multiple metabolic pathways were downregulated following cold acclimation (**Supplementary Figure S7**).

## Discussion

This study demonstrates that T-cell motility is highly sensitive to ambient temperature. At 37°C, T cells exhibited dynamic migratory behavior characterized by active lamellipodial extension and retraction. In contrast, motility progressively declined at temperatures below 32°C and was markedly reduced in non-acclimated cells at 26°C. Concomitantly, T cells adopted a rounded morphology, consistent with impaired actin cytoskeletal remodeling (5). Together, these findings indicate that temperature critically regulates T-cell dynamic behavior and cell–cell interactions (9, 18).

Remarkably, prior exposure to subphysiological temperatures induced cold acclimation, enabling T cells to regain motility even at low temperatures. Our results indicate that this adaptive response is associated with enhanced mitochondrial respiratory capacity—particularly increased spare respiratory capacity—rather than with changes in glycolytic activity. These findings suggest that metabolic remodeling, rather than glycolytic adaptation, underlies the functional recovery of T cells under cold conditions (25, 26, 29).

Notably, Seahorse assays were performed under conditions in which cells were immobilized on poly-D-lysine–coated culture wells, thereby minimizing ATP demand associated with cell migration. Because metabolic measurements were conducted under immobilized conditions, the observed increase in spare respiratory capacity reflects enhanced intrinsic respiratory reserve rather than ATP demand associated with active migration or T-cell–APC contact. Accordingly, basal oxygen consumption rate remained comparable between the two conditions, consistent with the reduced and relatively uniform energetic demand under these assay conditions. In addition, respiration reflects the balance of ATP turnover and mitochondrial efficiency, which may mask subtle metabolic differences. Taken together, we propose that metabolic remodeling during cold acclimation primarily enhances spare respiratory capacity, ensuring sufficient energy availability when demand arises under low-temperature conditions.

Importantly, cold acclimation was effective only within a restricted range of subphysiological temperatures and failed to occur at excessively low temperatures. Such threshold-dependent adaptations resemble stress-induced acclimation responses reported in other organisms (23, 30, 31). Notably, when evaluated at 37°C, cold-acclimated T cells exhibited reduced motility and respiratory activity compared with non-acclimated cells. This observation suggests that cold acclimation does not uniformly enhance cellular activity across the entire temperature range, but instead induces a cellular state optimized for cold environments, potentially at the expense of maximal functional capacity at physiological temperature.

Mechanistically, both actin and microtubule dynamics are highly temperature-sensitive. Intracellular microtubules are known to undergo rapid depolymerization when temperatures fall below physiological levels, reflecting the dependence of microtubule polymerization on GTP hydrolysis and environmental conditions (32, 33). Likewise, the polymerization rate of actin filaments decreases under low-temperature conditions, resulting in impaired actin turnover. Consistent with these observations, cold exposure has been shown to disrupt cytoskeletal remodeling in neurons (22). Together, these findings suggest that cytoskeletal instability is a major factor underlying the cell rounding and loss of motility observed at temperatures below 32°C. In contrast, cold acclimation may involve adaptive reprogramming that functionally stabilizes cytoskeletal dynamics, potentially in coordination with enhanced mitochondrial respiratory capacity, thereby enabling T cells to maintain dynamic morphology and migratory activity under cold conditions.

From a physiological perspective, immune cells are exposed to reduced temperatures in peripheral tissues as well as under conditions of clinical hypothermia. In addition, inflammation, ischemia, or tissue injury can lead to localized reductions in tissue perfusion, which may result in measurable decreases in regional temperature (34–36). At subphysiological temperatures, neutrophil motility, phagocytosis, and neutrophil extracellular trap (NET) formation are markedly reduced (37–39), and even mild perioperative hypothermia has been shown to increase the risk of surgical-site infections (19). Seasonal increases in respiratory infections have also been proposed to reflect impaired leukocyte activity in cooler airway tissues (40). Our findings that T-cell motility is suppressed under cold conditions are consistent with these previous observations. Importantly, however, our results further suggest that cold acclimation may serve as a protective adaptive mechanism that helps maintain T-cell functional competence in cold environments. Moreover, reduced temperature did not induce a significant loss of cell viability within 24 h, although short-term proliferative expansion was markedly suppressed, indicating that the observed functional alterations reflect adaptive state changes rather than nonspecific cytotoxic effects.

The temperature range examined in the present study (22–34°C) overlaps with several clinically and physiologically relevant conditions. Peripheral tissues such as skin and distal extremities frequently experience temperatures between 25°C and 34°C. Mild therapeutic hypothermia in clinical settings is typically maintained at 32–34°C, while moderate hypothermia may reach 28–32°C. In addition, airway epithelial surfaces can transiently cool below core body temperature during inhalation of cold air. Thus, the temperature conditions used in this study may approximate those encountered by immune cells in peripheral tissues and during hypothermia. These considerations support the physiological relevance of the observed temperature-dependent modulation of T-cell motility and metabolic adaptation.

Clinically, the concept of cold acclimation may provide a useful framework for understanding the vulnerability of distal hypothermic tissues—such as the hands, feet, and nasal mucosa—in cold environments, as well as the increased risk of infection associated with therapeutic hypothermia. These findings raise the possibility that enhancing or mimicking cold acclimation could help preserve immune competence in hypothermic patients. In addition, our results have important methodological implications. Standard protocols in cell physiology experiments often involve exposure to low temperatures for practical reasons, such as during cell handling or reagent preparation. Our findings suggest that such procedures may represent biologically relevant cold exposure capable of influencing subsequent functional assays, including measurements of cell motility and other temperature-sensitive cellular behaviors. Therefore, careful reporting of temperature conditions and, where feasible, minimization of subphysiological temperature exposure may improve experimental reproducibility without requiring substantial procedural changes.

Consistent with the observed reduction in short-term proliferative expansion at 26°C, transcriptomic analysis revealed downregulation of gene sets associated with DNA replication and cell-cycle progression. RNA sequencing analysis demonstrated extensive transcriptional reprogramming in cold-acclimated T cells, characterized by the upregulation of pathways related to ribosome biogenesis, RNA processing, transcriptional regulation, and protein quality control, accompanied by the downregulation of gene sets involved in DNA replication, DNA repair, and cell cycle progression. These coordinated changes suggest that cold-acclimated T cells transition from a proliferative and stress-responsive state toward a cellular state optimized for sustained transcriptional and translational activity. Notably, the suppression of gene sets associated with DNA replication and repair does not indicate increased genotoxic stress, but rather reflects a regulated downshift of proliferative programs. Such transcriptional reorganization is consistent with an adaptive remodeling process rather than an acute stress response and provides a plausible molecular basis for the altered migratory behavior observed following cold acclimation. Recent studies further support the concept that cold exposure elicits active and coordinated molecular adaptation rather than passive suppression of cellular activity. For example, transcriptomic analyses of hibernator-derived mammalian cells have identified glutathione peroxidase 4 (Gpx4) as a key mediator of cold resistance, highlighting the role of redox regulation in low-temperature survival (41). In addition, acute cold stress has been shown to activate the energy sensor AMPK while concomitantly suppressing mTORC1 signaling in skeletal muscle cells, suggesting a cell-autonomous reprogramming of energy utilization and protein synthesis pathways under cold conditions (42). These findings across distinct cell types reinforce the notion that low temperature triggers conserved adaptive signaling and transcriptional programs. Recent reviews further indicate that cold exposure elicits systemic metabolic adaptations, including enhanced mitochondrial biogenesis and thermogenic reprogramming in brown adipose tissue and skeletal muscle (43), supporting the concept of coordinated energy metabolism adjustment under low-temperature stress. In this context, the transcriptional remodeling observed in cold-acclimated T cells likely represents a coordinated adjustment of metabolic and biosynthetic pathways that supports functional adaptation to subphysiological environments. Although the precise upstream regulatory mechanisms remain to be elucidated, these findings support the concept that cold acclimation involves coordinated metabolic and transcriptional remodeling.

One limitation of the present study is that all analyses were performed using cultured T cells under controlled *in vitro* conditions. Although this approach allowed precise manipulation of temperature as well as detailed assessment of migratory behavior, metabolic activity, and transcriptomic remodeling, it does not fully recapitulate the complex thermal gradients and microenvironmental cues encountered by T cells *in vivo*. In addition, we did not perform molecular characterization of canonical immunological synapse structures; therefore, our conclusions focus primarily on temperature-dependent regulation of T-cell motility and associated metabolic and transcriptional changes. Metabolic measurements were conducted as representative experiments; although similar trends were reproducibly observed across independent cell preparations, additional fully replicated Seahorse analyses will be required to further validate the magnitude of the observed increase in mitochondrial spare respiratory capacity. Furthermore, the present study did not directly assess mitochondrial mass or the expression of oxidative phosphorylation (OxPhos) complexes. Therefore, whether the observed increase in spare respiratory capacity reflects enhanced mitochondrial abundance, altered respiratory complex expression, or changes in mitochondrial efficiency remains to be determined. Consequently, the molecular and functional alterations identified in this study should be interpreted within the context of the *in vitro* system. Nevertheless, these findings provide a foundation for future investigations into temperature-dependent regulation of immune cell behavior in physiological and pathological settings. Extending these analyses to *in vivo* models and tissue-specific immune responses will be essential for elucidating how cold acclimation contributes to immune regulation under naturally fluctuating thermal conditions.

In conclusion, our study demonstrates that T cells are highly sensitive to temperature but can adapt to cold environments through cold acclimation. This adaptive process restores T-cell motility at low temperatures and is accompanied by coordinated metabolic, cytoskeletal, and transcriptional remodeling. These findings indicate that temperature actively regulates immune cell behavior rather than serving solely as a passive environmental constraint. Collectively, our results provide a conceptual framework for understanding temperature-dependent regulation of immune cell dynamics under subphysiological conditions.

## Data Availability

The RNA sequencing data generated in this study have been deposited in the Gene Expression Omnibus (GEO) database under accession number GSE316736. All other data supporting the conclusions of this study are included in the article and its **Supplementary Material**.

## Supplementary material

Supplementary Figure 1 Geometrical definition of the cell–deformation area. The cell region at time t (green) and at t + 3 min (red) are superimposed. The cell–deformation area (yellow) was defined as the portion of the t + 3 min contour that lies outside the contour at time t, representing the newly occupied cellular area between consecutive time points. **(a)** Example in which the cell remains largely at the same position but undergoes shape remodeling. **(b)** Example in which the cell changes position while deforming. This parameter quantifies geometric contour remodeling and may increase during translational migration, spreading, or contraction depending on the relative gain and loss of area.

Supplementary Figure 2 Cell viability and proliferation under cold acclimation conditions. **(A)** Cell viability was assessed by trypan blue exclusion assay at 0 h and after 24 h incubation at 37°C or 26°C. Although a modest decrease in viability was observed after 24 h at 26°C, this difference did not reach statistical significance (Welch’s t-test, p = 0.0808). Data are presented as mean ± SD from three independent biological replicates (n = 3). For each biological replicate, cell counts were performed in quadruplicate and averaged prior to statistical analysis. **(B)** Cell proliferation over 24 h under the indicated temperature conditions. Proliferation was calculated as the fold change in total cell number relative to baseline (0 h). Cells cultured at 37°C exhibited robust expansion (2.45-fold increase), whereas proliferation was markedly suppressed at 26°C (1.066-fold increase). Data are presented as mean ± SD from three independent biological replicates (n = 3).

Supplementary Figure 3 ECAR profiles at 37°C in cold-acclimated and non-acclimated T cells. ECAR increased in both groups upon returning to 37°C, with a tendency toward higher activity in cold-acclimated cells. These results indicate that cold acclimation does not compromise glycolytic capacity and suggest that the respiratory advantage of acclimated cells is unlikely to be attributable to reduced glycolysis.

Supplementary Figure 4 Principal component analysis (PCA) of RNA-seq datasets. Principal component analysis was performed using normalized transcript counts from three independent biological replicates per condition (R1–R3). Each dot represents one biological replicate and is labeled directly on the plot. Blue dots indicate non-AC (non-acclimated, 37°C) T cells, and orange dots indicate AC (cold-acclimated, 26°C for 24 h) T cells. Biological replicates cluster tightly within each condition and are clearly separated along PC1. The percentage of variance explained by each principal component is indicated on the axes.

Supplementary Figure 5 Volcano plot of differentially expressed genes following cold acclimation. Volcano plot showing the global distribution of gene expression changes between non-acclimated (37°C) and cold-acclimated (26°C for 24 h) T cells. Each dot represents one gene. The x-axis indicates log2 fold change, and the y-axis indicates –log10(adjusted p-value). Genes meeting the predefined thresholds for differential expression (absolute log2 fold change ≥ 1 and adjusted p-value< 0.05) are highlighted in red (upregulated) and blue (downregulated), while non-significant genes are shown in gray.

Supplementary Figure 6 KEGG pathway enrichment analysis of differentially expressed genes following cold acclimation. **(a)** Significantly enriched KEGG pathways among upregulated genes in cold-acclimated T cells. **(b)** Significantly enriched KEGG pathways among downregulated genes. The x-axis indicates gene ratio, dot size represents the number of genes mapped to each pathway, and color indicates adjusted p-value (Benjamini–Hochberg correction). Pathways with adjusted p-value< 0.05 were considered significantly enriched.

Supplementary Figure 7 GO enrichment analysis (Biological Process) of genes downregulated in cold-acclimated T cells. The horizontal axis represents the −log10 of the adjusted p-values (FDR-corrected). Bar colors indicate the number of genes associated with each biological process. See also Supplementary Figure S5 for KEGG pathway enrichment analysis.

Supplementary Video 1 Comparison of T-cell behavior at 37°C and 26°C. Representative full-field time-lapse recordings of non–cold-acclimated T cells imaged at 37°C **(a)** and 26°C **(b)**. T cells were first seeded onto a glass-bottom dish and brought into focus prior to APC addition; therefore, cells initially in focus represent T cells. Images were acquired at 20-s intervals for 1200 s (total duration, 20 min) and are displayed at 200× playback speed (6 s per condition). A yellow asterisk indicates the T cell of interest during the first second of playback and fades out before the time-lapse sequence begins. Scale bar: 20 μm. No cropping or tracking overlays were applied.

Supplementary Video 2 High–temporal-resolution imaging of D10 T-cell motility and initial synapse formation (see **Figure 1**). Time-lapse images were acquired at 5-s intervals for 10 min and are displayed at 100× playback speed (6 s total duration). This recording highlights rapid lamellipodial dynamics and early immunological synapse formation at physiological temperature. Scale bar: 10 μm.

Supplementary Video 3 Effect of low temperature on T-cell motility and cell morphology (see also **Figure 2**). This video shows time-lapse imaging of T cells recorded at 20-s intervals over 10 min (30 frames) and converted into a 3-second movie (200× speed). T cells maintained at 37°C were imaged at 37°C **(a)**, 34°C **(b)**, 32°C **(c)**, 30°C **(d)**, 28°C **(e)**, and 26°C **(f)**. Scale bar: 10 μm **(a–f)**.

Supplementary Video 4 Activation of T-cell motility by cold acclimation (see also **Figure 3**). This video shows time-lapse imaging of T cells recorded at 20-s intervals over 10 min (30 frames) and converted into a 3-second movie (200× speed). T cells maintained at 37°C were imaged at 37°C **(a)** or acutely cooled to 26°C **(b)**, resulting in reduced motility at low temperature. In contrast, T cells acclimated to 26°C for 24 h exhibited restored motility when imaged at 37°C **(c)** and maintained motility even when imaged at 26°C **(d)**. Scale bar: 10 μm **(a–d)**.

Supplementary Video 5 Relationship between the duration of cold exposure and cold acclimation in T cells (see also **Figure 6**). This video shows time-lapse imaging of T cells recorded at 20-s intervals over 6.67 min (20 frames) and converted into a 2-second movie (200× speed). Panels d–j correspond to T cells imaged at 26°C after cold exposure for 3, 4, 6, 12, 18, 24, and 168 h, respectively, and illustrate the progressive changes in cell motility and morphology associated with cold acclimation. Scale bar: 10 μm (d–j).
