# Supplementary material for: Adaptive restoration of T-cell motility by cold acclimation through metabolic and transcriptional remodeling
Source: Front Immunol. 2026 Apr 10;17:1789182. doi: 10.3389/fimmu.2026.1789182 (PMC13147160; doi:10.3389/fimmu.2026.1789182)
Supplement: Supplementary Figure 1 — Geometrical definition of the cell–deformation area. The cell region at time t (green) and at t + 3 min (red) are superimposed. The cell–deformation area (yellow) was defined as the portion of the t + 3 min contour that lies outside the contour at time t, representing the newly occupied cellular area between consecutive time points. (a) Example in which the cell remains largely at the same position but undergoes shape remodeling. (b) Example in which the cell changes position while deforming. This parameter quantifies geometric contour remodeling and may increase during translational migration, spreading, or contraction depending on the relative gain and loss of area. [file DataSheet1.zip › Supplementary_Figures_S1-S7.pdf]

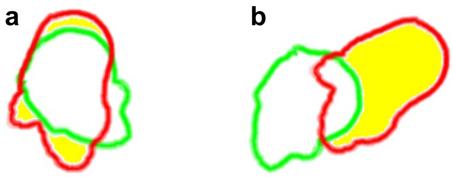

Supplementary Figure S1. Geometrical definition of the cell–deformation area. The cell region at time t (green) and at t + 3 min (red) are superimposed. The cell–deformation area (yellow) was defined as the portion of the t + 3 min contour that lies outside the contour at time t, representing the newly occupied cellular area between consecutive time points. (a) Example in which the cell remains largely at the same position but undergoes shape remodeling. (b) Example in which the cell changes position while deforming. This parameter quantifies geometric contour remodeling and may increase during translational migration, spreading, or contraction depending on the relative gain and loss of area.

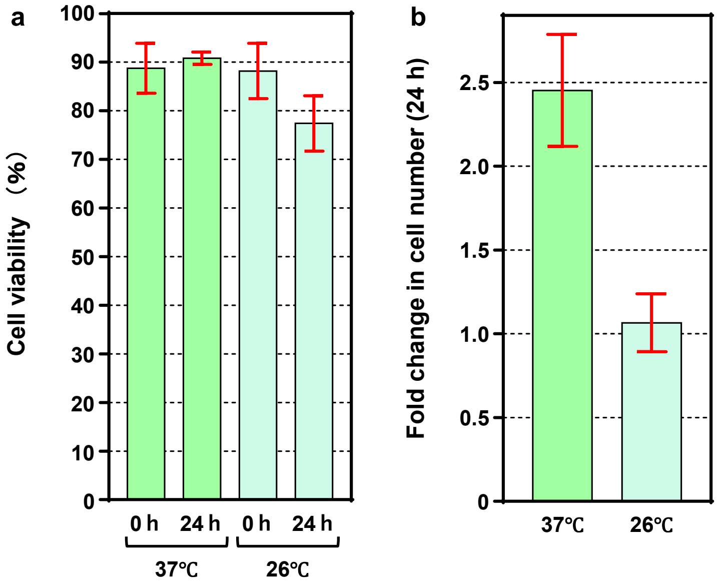

Supplementary Figure S2. Cell viability and proliferation under cold acclimation conditions. (A) Cell viability was assessed by trypan blue exclusion assay at 0 h and after 24 h incubation at 37°C or 26°C. Although a modest decrease in viability was observed after 24 h at 26°C, this difference did not reach statistical significance (Welch's t-test, $p = 0.0808$). Data are presented as mean ± SD from three independent biological replicates ($n = 3$). For each biological replicate, cell counts were performed in quadruplicate and averaged prior to statistical analysis. (B) Cell proliferation over 24 h under the indicated temperature conditions. Proliferation was calculated as the fold change in total cell number relative to baseline (0 h). Cells cultured at 37°C exhibited obust expansion (2.45-fold increase), whereas proliferation was markedly suppressed at 26°C (1.066-fold increase). Data are presented as mean ± SD from three independent biological replicates ($n = 3$).

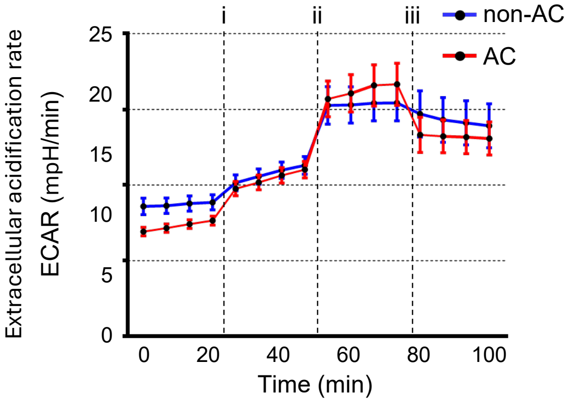

Supplementary Figure S3. ECAR profiles at 37°C in cold-acclimated and non-acclimated T cells. ECAR increased in both groups upon returning to 37°C, with a tendency toward higher activity in cold-acclimated cells. These results indicate that cold acclimation does not compromise glycolytic capacity and suggest that the respiratory advantage of acclimated cells is unlikely to be attributable to reduced glycolysis.

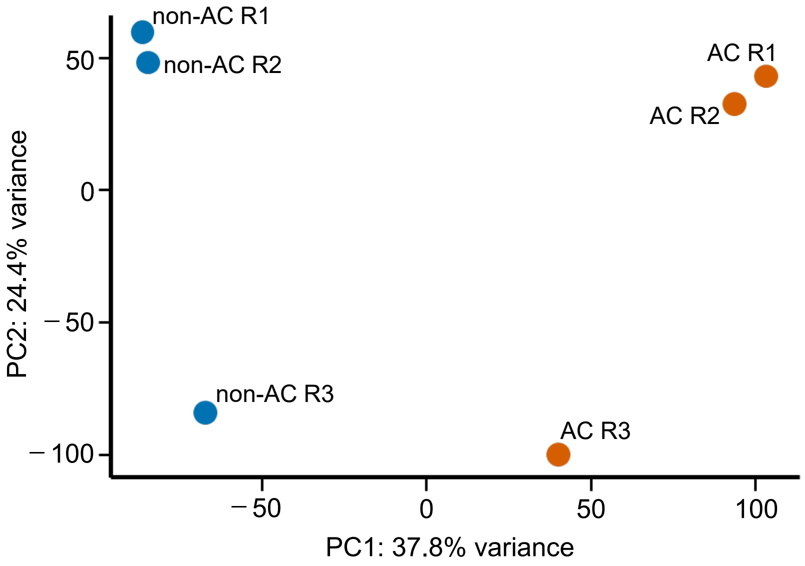

Supplementary Figure S4. Principal component analysis (PCA) of RNA-seq datasets. Principal component analysis was performed using normalized transcript counts from three independent biological replicates per condition (R1–R3). Each dot represents one biological replicate and is labeled directly on the plot. Blue dots indicate non-AC (non-acclimated, 37°C) T cells, and orange dots indicate AC (cold-acclimated, 26°C for 24 h) T cells. Biological replicates cluster tightly within each condition and are clearly separated along PC1. The percentage of variance explained by each principal component is indicated on the axes.

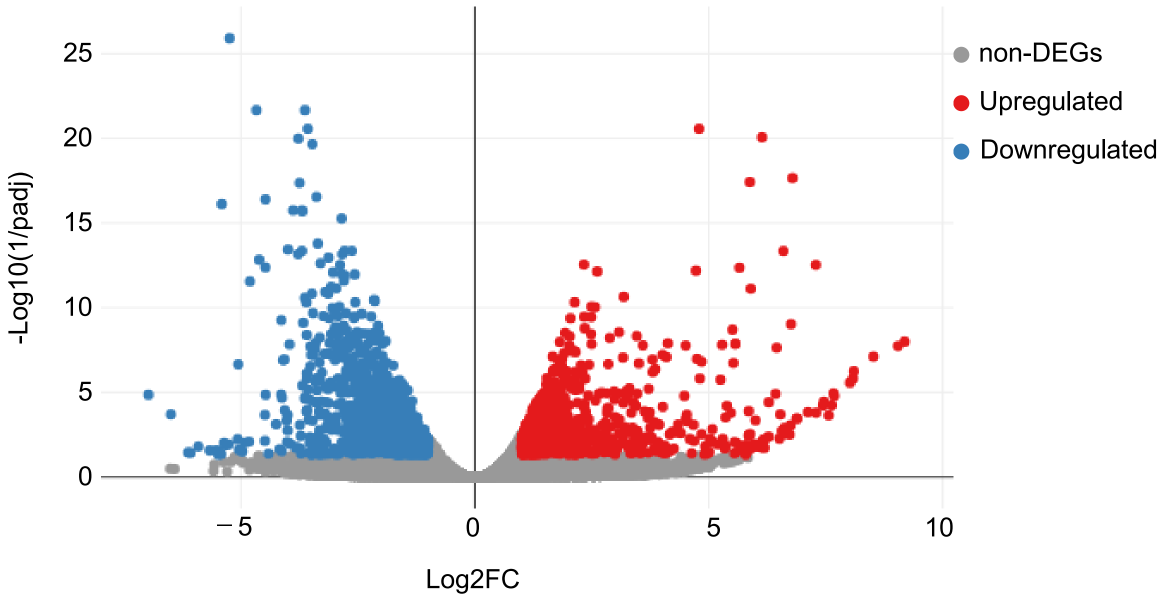

Supplementary Figure S5. Volcano plot of differentially expressed genes following cold acclimation. Volcano plot showing the global distribution of gene expression changes between non-acclimated (37°C) and cold-acclimated (26°C for 24 h) T cells. Each dot represents one gene. The x-axis indicates log2 fold change, and the y-axis indicates –log10(adjusted p-value). Genes meeting the predefined thresholds for differential expression (absolute log2 fold change ≥ 1 and adjusted p-value< 0.05) are highlighted in red (upregulated) and blue (downregulated), while non-significant genes are shown in gray.

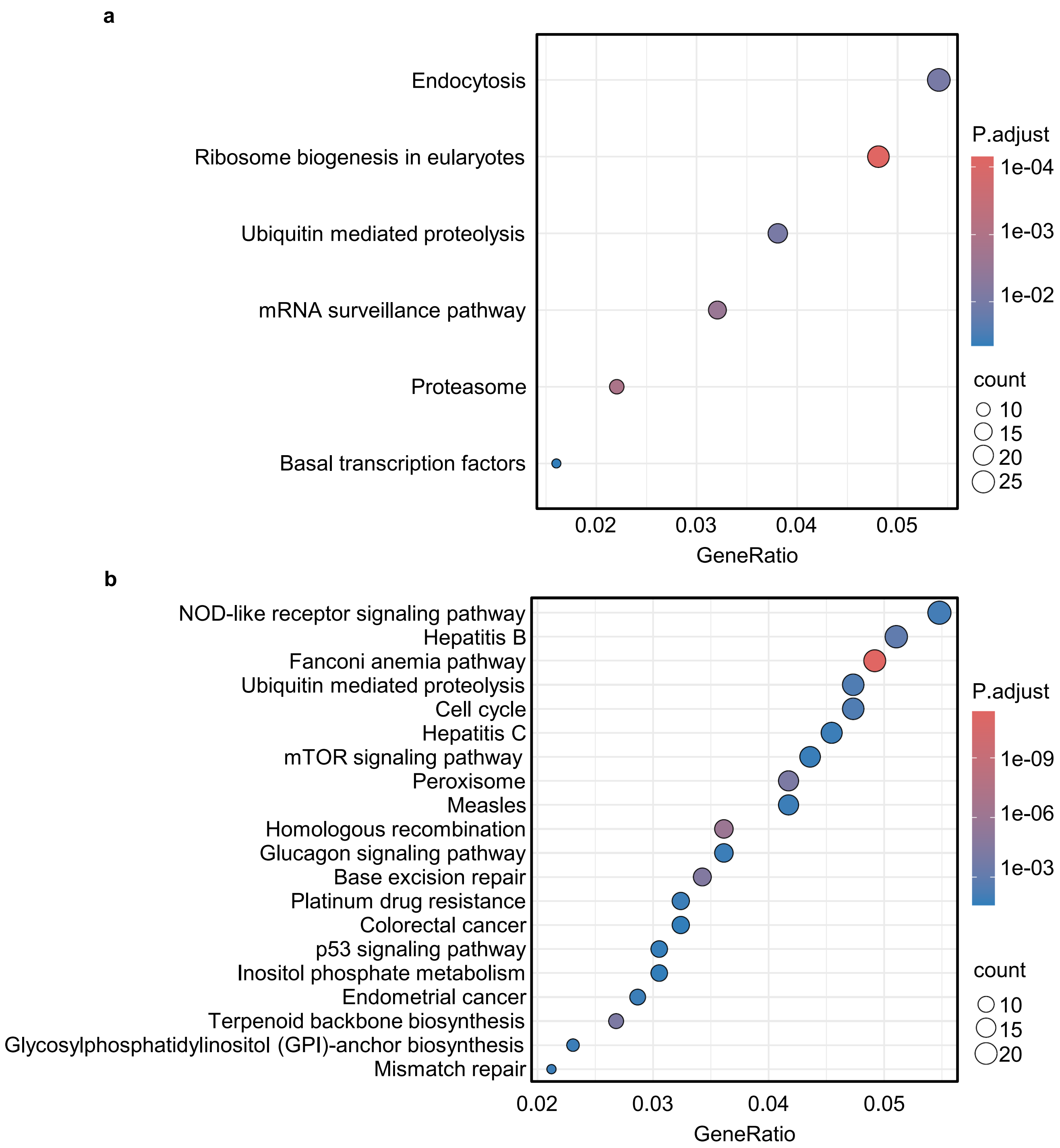

Supplementary Figure S6. KEGG pathway enrichment analysis of differentially expressed genes following cold acclimation. (a) Significantly enriched KEGG pathways among upregulated genes in cold-acclimated T cells. (b) Significantly enriched KEGG pathways among downregulated genes. The x-axis indicates gene ratio, dot size represents the number of genes mapped to each pathway, and color indicates adjusted p-value (Benjamini–Hochberg correction). Pathways with adjusted p-value< 0.05 were considered significantly enriched.

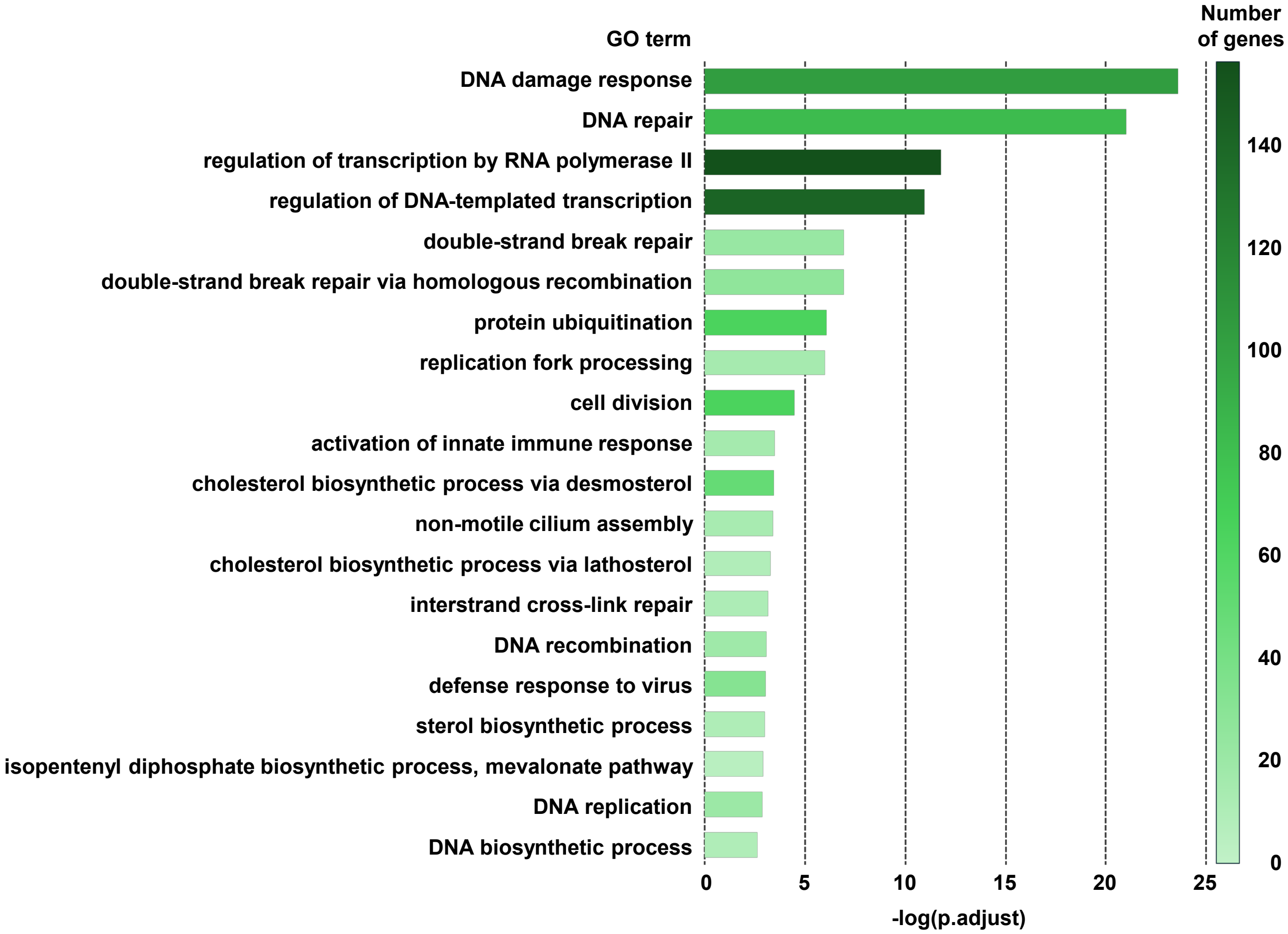

Supplementary Figure S7. GO enrichment analysis (Biological Process) of genes downregulated in coldacclimated T cells. The horizontal axis represents the −log10 of the adjusted p-values (FDR-corrected). Bar colors indicate the number of genes associated with each biological process. See also Supplementary Figure S5 for KEGG pathway enrichment analysis.
